# The Different Facets of Heart Rate Variability in Obstructive Sleep Apnea

**DOI:** 10.3389/fpsyt.2021.642333

**Published:** 2021-07-22

**Authors:** Hua Qin, Nicolas Steenbergen, Martin Glos, Niels Wessel, Jan F. Kraemer, Fernando Vaquerizo-Villar, Thomas Penzel

**Affiliations:** ^1^Interdisciplinary Center of Sleep Medicine, Charité-Universitätsmedizin Berlin, Berlin, Germany; ^2^Imperial College London School of Medicine, London, United Kingdom; ^3^Department of Physics, Humboldt Universität zu Berlin, Berlin, Germany; ^4^Biomedical Engineering Group, Universidad de Valladolid, Valladolid, Spain; ^5^Centro de Investigación Biomédica en Red-Bioingeniería, Biomateriales y Nanomedicina, Valladolid, Spain; ^6^Saratov State University, Russian Federation, Saratov, Russia

**Keywords:** obstructive sleep apnea, heart rate variability, autonomic dysfunction, central autonomic networks, time-window analysis, time-domain analysis, frequency-domain analysis, non-linear analysis

## Abstract

Obstructive sleep apnea (OSA), a heterogeneous and multifactorial sleep related breathing disorder with high prevalence, is a recognized risk factor for cardiovascular morbidity and mortality. Autonomic dysfunction leads to adverse cardiovascular outcomes in diverse pathways. Heart rate is a complex physiological process involving neurovisceral networks and relative regulatory mechanisms such as thermoregulation, renin-angiotensin-aldosterone mechanisms, and metabolic mechanisms. Heart rate variability (HRV) is considered as a reliable and non-invasive measure of autonomic modulation response and adaptation to endogenous and exogenous stimuli. HRV measures may add a new dimension to help understand the interplay between cardiac and nervous system involvement in OSA. The aim of this review is to introduce the various applications of HRV in different aspects of OSA to examine the impaired neuro-cardiac modulation. More specifically, the topics covered include: HRV time windows, sleep staging, arousal, sleepiness, hypoxia, mental illness, and mortality and morbidity. All of these aspects show pathways in the clinical implementation of HRV to screen, diagnose, classify, and predict patients as a reasonable and more convenient alternative to current measures.

## Introduction

Obstructive sleep apnea (OSA) is closely associated with neurocognitive, behavioral, psychophysiological states, and cardiovascular outcomes (1–3). It is estimated that globally ~1 billion adults have mild to severe sleep apnea. Some countries have a prevalence over 50% and it still is increasing. The consequent health and financial burden can be minimized by effective diagnosis and treatment (4). To that effect, recently, the role of cardiovascular autonomic dysfunction has received increasing attention as an independent risk factor for clinical complications in OSA (5). Heart rate variability (HRV) has been generally accepted as a non-invasive tool to quantify cardiovascular autonomic modulation under varying healthy and pathogenic conditions (6, 7). HRV measures the variation between beat-to-beat intervals over a time series (6). It is an integrated reflection of central-peripheral neural feedback mechanisms to the heart *via* mediating sympathovagal inflow and outflow (8). Previous studies suggested that in conjunction with brain imaging, HRV analysis has been used to investigate the connection between autonomic cardiac modulation and sleeping brain activity (9).

Currently, HRV analysis, including time-domain, frequency-domain, and non-linear analysis, is used to explore the activities of sympathetic and parasympathetic nervous systems (6, 10). Time-domain analysis quantifies the magnitudes of variation. The most relevant time-domain parameters are described in Table 1. For example, the standard deviation of normal-to-normal intervals (SDNN), a global HRV metric, is frequently used as a prognostic indicator of cardiovascular risk in different populations (11). Frequency-domain analysis is used for partitioning the rhythms of electrocardiography (ECG) signals into different frequencies (12, 13). This analysis helps gain a better understanding of cardiac control as ECG frequencies could be related to intrinsic elements modulated by the cardiac autonomic system alone. Power spectral density (PSD) is the standard method employed to estimate the distribution of the HRV signal power over frequency. Table 2 shows the main frequency-domain parameters typically computed from the PSD of HRV (14). High frequency (HF) components mainly present parasympathetic activity. However, there is a disagreement with regards to the low frequency (LF) components. Some studies suggested that LF, when expressed in normalized units, is a quantitative marker of sympathetic modulation, but other studies view LF as a reflection of both sympathetic and vagal activity mainly mediated by the baroreflex. Thus, the LF/HF ratio is considered a detection index for either sympathovagal balance or sympathetic modulations (15). Apart from the conventional PSD, other frequency-domain methods are also used to analyze the frequency content of the HRV, such as high order spectral analysis and wavelet analysis. Non-linear HRV captures dynamic sequences of the heartbeat time series related to randomness and self-similarity (10, 16). It is suggested that non-linear fluctuations result from interactions of electrophysiological, hemodynamic, and humoral variables, as well as by autonomic and central nervous regulation (17). Pathologically monotonous and erratic HRV patterns are associated with negative outcomes in cardiac patients (18). OSA patients show a reduced dynamic complexity (19, 20). The clinical relevance of non-linear HRV in OSA still needs to be the established. Table 3 summarizes the reported non-linear parameters and methods in current studies on HRV (6, 14, 21). However, this not by any means an exhaustive list.

**Table 1:** Selected time-domain HRV measures.

**Variable**	**Units**	**Definition**
**Time-domain analysis**		
SDNN	ms	Standard deviation of normal to normal (NN) interval time series
SDANN*X (X = 1, 5)*	ms	Standard deviation of BBI averages in successive X-minute intervals
RMSSD	ms	Square root of the mean squared differences of successive NN intervals
pNN*X (X = 50, 100, 200)*	%	NN>Xms counts divided by the total number of all NN intervals.
pNNl*X (X = 10, 20, 30)*	%	NN < Xms counts divided by the total number of all NN intervals.
**Time-domain geometric measures**		
HRVi	–	HRV triangular index
TINN	ms	Baseline width of the minimum square difference triangular interpolation of the NN interval histogram

**Table 2:** Selected frequency-domain HRV parameters.

**Variable**	**Units**	**Definition**
**Frequency-domain analysis**		
TP	ms^2^	Total power (0–0.4 Hz)
ULF	ms^2^	Ultra-low frequency (0–0.01 Hz)
VLF	ms^2^	Very low frequency (0.01–0.04 Hz)
LF	ms^2^	Low frequency (0.04–0.15 Hz)
HF	ms^2^	High frequency (0.15–0.4 Hz)
LF/HF	–	Ratio of LF to HF
HF nu	–	Normalized high frequency power HF/(LF+HF) × 100
LF nu	–	Normalized low frequency power LF/(LF+HF) × 100

**Table 3:** Selected non-linear HRV parameters and methods.

**Variable**	**Units**	**Definition**
**Chaotic invariant analysis**		
D_2_	–	Correlation dimension
LLE	–	Largest Lyapunov exponent
FD	–	Fractal dimension
H	–	Hurst exponent
**Poincare plots**		
SD1	ms	Standard deviation around the Y-axis of the Poincaré plot
SD2	ms	Standard deviation around the X-axis of the Poincaré plot
**Detrended fluctuation analysis (DFA)**		
α_1_	–	Slope of the short-time scales of the DFA profile
α_2_	–	Slope of the long-time scales of the DFA profile
**Entropy analysis**		
ApEn	–	Approximate entropy
SampEn	–	Sample entropy
RenyiEn	–	Renyi entropy
ShanEn		Shannon entropy
REEn	–	Renormalized entropy
**Recurrence plots (RP)**		
MDL	–	Average length of diagonal lines in RP
TT	–	Average length of vertical lines in RP
DET	–	Rercentage of recurrent points forming diagonal lines in a RP
LAM	–	Rercentage of recurrent points forming vertical lines in a RP
ENTR	–	Shannon entropy of the distribution of diagonal lines in a RP
**Symbolic dynamics**		
Fwshannon	–	Shannon entropy of the probabilities of occurrence of the words of the symbol sequence
Forbword	–	Number of words of length 3 that never or only seldom occur
Wsdavar	–	Standard deviation of the word sequence
Phvar5	–	Portion of high-variability patterns in the NN interval time series (>5ms)
Plvar20	–	Portion of low-variability patterns in the NN interval time series (<20ms)
WpsumXY (XY = 02, 13)	–	Percentage of words which contain the symbols “X” and “Y”

Heart rate and blood pressure oscillations are characterized by parasympathetic predominance and sympathetic inhibition in normal subjects during non-rapid eye movement (NREM) sleep (22). In contrast, sympathetic predominance and parasympathetic withdrawals are found during similar rapid eye movement (REM) sleep and wakefulness. As a result, there is a reduction of heart rate and blood pressure during NREM sleep and an increase during REM sleep. However, patients with OSA manifest a heterogeneous pathophysiology (e.g., upper airway anatomical collapsibility, loop gain, arousal threshold, and upper airway gain) and characteristics (e.g., recurrent apnea and hypopnea, nocturnal hypoxemia, frequent awakenings, and daytime sleepiness) (23). Consequently, hypoxia and arousal in OSA are thought to potentially be the main factors leading to certain hemodynamic instability, causing fluctuations in heart rate that contribute to the changes in HRV. Previous studies have shown the detrimental effect of OSA on HRV either during wakefulness or sleep (24–26), suggesting a relationship between OSA severity and cardiovascular autonomic modulations using conventional HRV analysis. Additionally, (27). suggested that prolonged alterations in autonomic function existed even in snoring subjects. Those findings highlighted the potential cumulative impacts of OSA on HRV. On the other hand, Idiaquez et al. (28) found independent pathophysiological mechanisms may underlie the modulation of neurobehavioral changes and HRV in OSA despite sharing common cerebral control regions and mediated pathways. Although the HRV time window is more related to mathematics, physics and statistics, its determination in OSA-related events (e.g., sleep apnea, arousal, and periodic limb movement) is crucial in reflecting the relationship between autonomic changes and OSA-related physiological changes. Furthermore, it allows for the discovery of how the cardiovascular, respiratory, autonomic, and central nervous systems interact with each other in OSA. The HRV time window is also particularly important in coupling analysis such as synchronization and ensemble symbolic coupling, potentially revealing direction and strength of dynamic cardiovascular transition (29, 30).

Taken together, HRV could provide a static and a dynamic perspective to observe the changes in connectivity between central and cardiac autonomic modulation during sleep and its persistent influence during daytime. This review focuses on neuro-cardiac autonomic regulatory mechanisms and the multifaceted applications of HRV in OSA as a potential additional clinical diagnostic tool.

## Time-Window Analysis Technology of HRV

HRV is usually measured over a short-term (5–15 min) or long-term period (1–24 h). Long-term measurements are generally used to assess mortality and adverse prognosis of patients, but short-term measurements have been shown to be sufficiently stable and applicable for screening. However, 5-min recordings only had strong correlation with HF (31). Li et al. (32) assessed short-term analysis to be suitable for estimation of autonomic status and tracking dynamic changes but long-term changes to be better as an autonomic function assessor and prognostic indicator. The issue is that the cardiovascular system is in constant flux and thus HRV parameters constantly fluctuate at rest or during various conditions (33–35). The selection of the time window is thus a crucial aspect in HRV analysis (36, 37).

Most studies use short-term time windows with their analytic techniques; 2–5 min with Fast Fourier Transform (FFT) or autoregression, or 1–2 min with multiple trigonometric regressive spectral (MTRS) analysis (6, 38, 39). New techniques such as short time Fourier transform or Wigner-Ville transforms (WVT) are able to return instant power spectral profiles (40, 41). Short-term windows have the advantages of being easy to perform, easy to control for confounding factors, require the least data processing and describe dynamic HRV changes in short time periods (32). However, the constant flux of HRV values means that it may not be stable and that it cannot measure long RRI fluctuations, especially the ultra-LF (6, 37). Ultra-short term HRV has shown potential for diagnostic capability within a short timespan immediately after an apneic event (e.g., arousals). However, it is only able to measure time-domain parameters and no frequency-domain parameters, severely limiting its informational output and, like short-term HRV, the constant flux may mean it is unstable (42).

Longer time windows are commonly analyzed with FFT or autoregression, as they are commonly divided into 1–5-min periods and averaged to provide a mean for the total time segment (6, 36, 37). Alternatively, the entire time window is used as a single data segment, which yields similar results for LF and HF over 24 h (43). Its primary advantage is in collecting stable and reflective ECG data over an extended period of time. Any singular 5-min window can vary wildly from another, and thus measuring HRV over a whole day allows for better estimations of fluctuations (32). However, long-term HRV analysis is financially expensive and labor intensive, on top of requiring more considerations about filtering and analysis (6, 37).

Li et al. (32) suggest three main uses of HRV analysis: evaluating autonomic function in specific populations, describing changes in autonomic function, and prognosis. To evaluate autonomic function in specific conditions such as myocardial infarction (MI), hypertension and Parkinson's, short-term analysis may be used (44–47). Long-term analysis can be used for daytime or sleep analysis, or a full 24-h analysis and is thus more suitable for assessing OSA autonomic status, in line with what most studies use. Although HRV analyses of different window lengths are closely correlated, they do not always align (48–50). Studies in this particular area are particularly lacking and require further investigation. In describing change in autonomic function, both short-term and long-term analysis can be used over a period of hours or months, whereas short-term can measure changes in minutes. In this regard, measuring changes due to apneic episodes is a useful application of short-term analysis. However, this type of short-term analysis likely already falls under an overnight long-term analysis (32). Many OSA studies use overnight HRV with 5-min time windows. Still, more studies are needed directly comparing the two with respect to OSA. Using HRV as a prognostic indicator is usually done *via* long-term analysis. Many studies assessing mortality have used overnight or 24-h HRV analyses to obtain a reliable prognosis and use 5-min windows within these time periods to compare HRV (49–55).

It is clear that the majority studies use long-term HRV analysis for the assessment of OSA, mostly with time-frequency domains. However, whether this is the best use of HRV is not clear as there is a lack of studies reporting on this particular aspect. To further this point, there is no agreement on a single method with which to analyze HRV in sleep apnea as a wide variety of time windows within an overnight sleep study are analyzed in the literature. Studies aimed at short-term changes potentially analyze 2-min epochs around apneas and hypopneas or arousal-free windows or look at the first and last 10-min segments during SDB and stable breathing during NREM, for example (56–58). Long-term analysis aimed studies sometimes look at averaged consecutive 5-min windows in different sleep stages (stage 2 is commonly used as a reflection of NREM sleep) or stable 5-min intervals from each sleep stage or the first 5-min segment of each sleep stage, to name a few (59). The standardization of time window approach to provide a regulated and agreed upon methodology of time window analysis that presents comparable and valuable ECG changes in OSA during an overnight sleep study is an area in pressing need of further study. Although time window analysis is a potent area of research to solidify first, the current use of HRV has shown promise and accuracy in many areas, from prognosis to sleep stage detection.

## Technical Features of HRV Measurements

There are some important technical features that affect HRV analysis. In this respect, ECG sampling rate could be critical to the accuracy and reliability of the HRV time series. Two hundred and fifty hertz or higher are recommended, however, given the minor relative errors among various ECG sampling rates, over 100 Hz are acceptable in time-domain, frequency-domain, and non-linear HRV analysis (60–62). Concerning the extraction of RR intervals, there is a big variety of algorithms aimed at detecting the R peaks (63), being the Pan and Tompkins the most well-known one (64). However, artifacts and ectopic beats are usually present in ECG recordings, which can result in non-normal RR intervals, thus affecting HRV analysis. This issue is addressed by detecting and correcting non-normal beats. The detection of non-normal beats can be performed using different automatic methods: time and morphological approaches, methods based on the morphological transformation, wavelet-based approaches, empirical mode decomposition methods, and neural network approaches (65). Conversely, deletion, interpolation (zero-degree, linear interpolation, and cubic spline methods), and adaptive approaches are used to correct non-normal beats (65). However, these methods can also cause measurement errors in the HRV signal, which demands more research efforts on the development of correction methods.

## Influence of Sleep Structure on HRV

According to the American Academy of Sleep Medicine (AASM), sleep is categorized into non-rapid eye movement (NREM) stages N1, N2, N3, into stage rapid eye movement sleep (REM), and into stage Wake by visual electroencephalogram (EEG), electrooculogram (EOG), and chin electromyogram (EMG) scoring (66). Collectively, studies have reported a general trend in HRV during healthy sleep; LF and the LF/HF ratio are high in Wake and decrease in NREM sleep, peaking once more during REM sleep, while HF follows the opposite trend (67–71). This corresponds to muscle sympathetic and parasympathetic activity observed in sleep (72, 73). Opposingly, Ako et al. (74) reported decreasing LF and LF/HF ratio during NREM and an increase during REM but no differentiation of HF during the NREM and REM stages in healthy sleep. However, Abdullah et al. (75) reported a strong correlation between EEG delta, sigma, and beta bands with HRV parameters (LF, HF, LF/HF ratio). Jurysta et al. (76) and Köhler and Schönhofer (77) reported negative correlations between cardiac vagal predominance and delta sleep EEG and abnormalities in the respective power bands. In contrast, Yang et al. (71) reported a negative relation between cardiac sympathetic regulation and depth of sleep, but not vagal regulation. The repeatability of the measurements in HRV parameter patterns in relation to the sleep stages, however, certifies the suggested physiological activity seen during sleep.

## Influence of Sleep Apnea on HRV During Daytime

OSA seems to have long-term effect on HRV even during wakefulness with the absence of sleep apnea. Limited data regarding its underlying mechanisms during daytime or ambulatory wake state is reported. It is assumed that autonomic dysfunction plays a key role in persistent OSA related outcomes, leading to a blunted diurnal HRV pattern. Using 10-min ECG segments and muscle sympathetic nerve activity (MSNA) recordings during daytime, Narkiewicz et al. found that the magnitude of cardiovascular variability is associated with the severity of OSA. There was reduced RR variance, increased sympathetic tone and decreased parasympathetic tone in moderate-to-severe OSA populations compared to matched controls (25). Balachandran et al. (78) found significantly different LF, HF, and LF/HF between mild OSA without any symptoms and healthy controls in waking condition. Similarly, Hilton et al. (79) found that at daytime amount of HF power as marker of vagal activity is negatively correlated with the apnea-hypopnea index (AHI) and %HF and LF/HF were shown to be different in OSA patients compared to controls. Respiratory sinus arrhythmia (RSA) is a natural physiological phenomenon reflecting cardiopulmonary coupling characterized by periodic increases and decreases with heartbeat synchronized with respiration, whereby heartbeat increases during inspiration and decreases during expiration. Consequently, normal respiration HRV is different than deep respiration HRV and apneic respiration HRV due to the inspiration-expiration pattern (80). Given the altering effect of respiration on HRV, Khoo et al. (81) developed two modified spectral HRV measures (the modified LF/HF and the average gain relating respiration to RR changes) to show cardiac autonomic alternations in OSA and non-OSA during in relaxed wakefulness and stage 2 sleep compared to standard spectral metrics. They found that the modified spectral HRV measures are more sensitive than the traditional measures, suggesting a respiration–correlated component should be considered in HRV analysis. In addition, Wang et al. (24) suggested that autonomic dysfunction was related to OSA severity. However, they mainly evaluated gender differences in frequency-domain HRV measures, rather than with different levels of severity of OSA, showing significantly higher LF in male patients from wakefulness to sleep state. Park et al. (82) examined the correlation between severity of OSA and overnight HRV during wakefulness in moderate/severe OSA. They found increased total power (TP), LF, LF/HF, and HRV triangular index in the severe group compared to the moderate one. Comparably, Qin et al. (83) found a significant relationship between 5-min HRV measures during wakefulness prior to sleep onset and OSA severity in a large international clinical cohort, suggesting reduced time-domain and non-linear HRV measurements in severe OSA compared to other AHI groups. Moreover, their findings demonstrated that OSA seems to play a significant role in obese patients, showing a shift to sympathetic predominance only in obese patients with more severe OSA with increased LF and higher LF/HF compared to obese patients without OSA. There are also hints that OSA therapy normalizes autonomic balance not only during sleep but also at daytime. Glos et al. (84) found that both continuous positive airway pressure (CPAP) as well as mandibular advancement therapy (MAD) therapy led to increased vagal output to the heart, indicated by increased HRV HF components calculated from 5-min short-time recordings under conditions of controlled breathing at daytime.

## Influence of Sleep Apnea on HRV During Sleep

**Figure 1:**
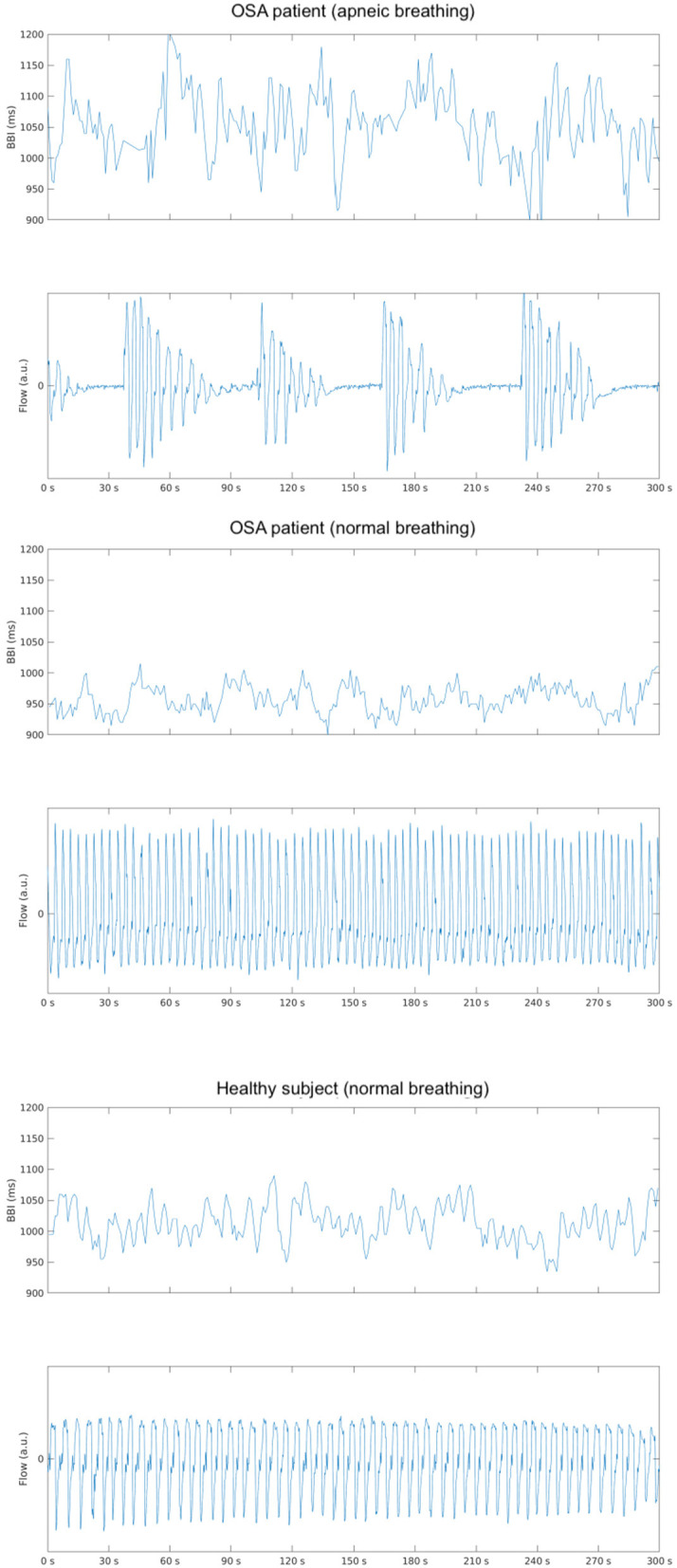
Depicts an example of the changes in beat-to-beat intervals (BBI) in an obstructive sleep apnea (OSA) subject with **(upper)** and without **(middle)** the presence of apneic events and a healthy subject **(bottom)** during stage 3 sleep in the supine position.

The normalizing effect of OSA therapy on HRV during sleep has also been suggested. Earlier studies report higher sympathetic activity during wake and sleep, but this has normalized, perhaps because of CPAP (73, 85). This is supported by Noda et al.'s (86) study reporting that managed OSA and better sleep quality was associated with a decreased LF. Since then, Abdullah et al. (75) reported an increase in LF and LF/HF in Stages 2 and 3 in sleep apnea compared with healthy patients. This corresponds with results from Dingli et al. (56) and Jurysta et al. (87), which showed an increase in sympathetic and decrease in parasympathetic activity during NREM apnea episodes. Bonnet and Arand (67) reported EEG arousal during Stage 2 and associated HRV changes. Palma et al. reported OSA with hypoxia patients had increased LF and LF/HF during N1 and N2 and REM compared to OSA without hypoxia patients and controls. They also reported that OSA with and without hypoxia had lower HF during NREM and REM in compared to controls (88). In contrast, Jurysta et al. reported no changes in LF/HF and RRI between healthy and OSA subjects. They did however suggest that sympathetic and vagal surges during apneic episodes may suppress the normal shifts between stages of sleep (76). Trimer et al. reported higher LF and LF/HF in moderate OSA subjects compared to normal subjects. Mild OSA subjects also failed to show the linear HRV difference between sleep stages present in non-OSA subjects (20). Kesek et al. studied the relationship between OSA severity and HRV in 387 women and found that high AHI was associated with low variation of sympathetic activity between REM and NREM, suggesting a depressed sympathetic drive and a disability increasing it during REM. These results differ from others, but the study was in healthy women only and gender differences in HRV have been reported (89). Reynolds et al. found a positive correlation between apnea severity and LF in wakefulness and REM sleep, but LF was lower in those with a higher BMI during REM sleep in 105 OSA patients. The suggestion is thus that there is possible autonomic dysfunction in obese apnea patients (90). On the contrary, Oh et al. (91) conducted a 27-participant study and concluded that OSA during REM sleep is not a major contributor of autonomic dysfunction. However, the study was conducted on a small cohort and requires repeated testing to confirm results. In addition, Lado et al. (92) found significant differences in spectral HRV in all three types of intervals (normal breathing, borderline episodes, and sleep apnea) among non-OSA control, mild, and severe OSA subjects during sleep, suggesting that patients with OSA have reduced HRV during sleep even without the presence of sleep apnea (Figure 1). In addition, Szollosi et al. (58) compared HRV patterns between OSA and central sleep apnea (CSA), finding higher very low frequency (VLF) percentage, lower LF percentage and HF percentage in CSA, while no significant changes during normal breathings between patients with OSA and CSA. Their results suggested that CSA and OSA have different autonomic modulation, respectively. Overall, the research presented shows increased sympathetic activity during apneic sleep with episodic surges in comparison to healthy sleep, reflected *via* increased LF and LF/HF parameters in HRV.

**Figure 2:**
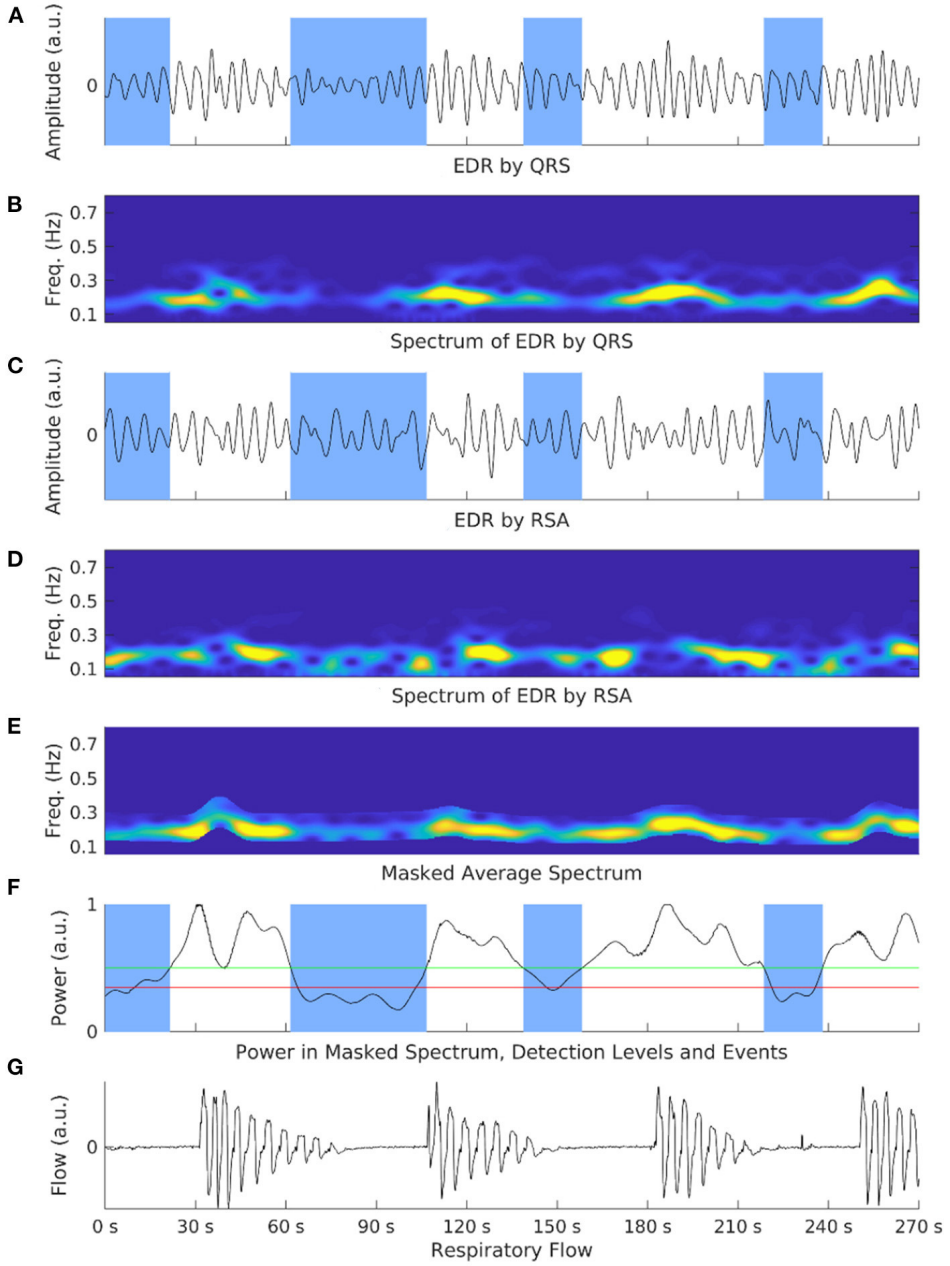
Shows an exemplary illustration of the respiratory power index (RPI) and electrocardiograph-derived respiration (EDR) methods in an OSA patient. Overnight electrocardiograph recordings are processed and cut into limited time segments. EDR signals are calculated *via* ECG respiration embeddings such as QRS complex **(A)** or respiratory sinus arrhythmia (RSA) **(C)**. Spectrograms of both embeddings are also generated **(B,D)**. These spectrograms are normalized and averaged to amplify the respiration-based component and mask non-respiration-related power **(E)**. The power is calculated at each step with two selection events **(F)**. A respiratory flow shows corresponding events to the power spectrum **(G)**. The number of detected apneic events is the RPI.

In seeing the relation of parameters to apneic sleep, there appears to be potential in using HRV as a cost-effective tool for the detection of apnea. Some studies report that cardiac changes visibly precede EEG changes with a range of 10 beats to 5 min in apneic episodes (67, 76). Penzel et al. (93) reported that it was possible to classify apnea *via* HRV with 100% accuracy when comparing to normal subjects and 90% when comparing normal and apneic minute intervals in 35 samples. Roche et al. (94) reached sensitivities of 83 and 89.7% and specificities of 98.1 and 96.5% when using SDNN as a marker in the detection of OSA in groups of 91 and 52 patients, respectively. Then using wavelet decomposition parameters in 147 patients, Roche et al. (95) reached a sensitivity of 92.4% and specificity of 90.1%. Karasulu et al. (96) found a 90.4% sensitivity and 50% specificity when using a VLF cut-off of 9.12, 80 and 76.2% when SDNN was higher than 83 and 73.3 and 85.7% with an SDNN cut-off of 62 in 87 patients. Offering a variant to these results, Abdullah et al. combined EEG and HRV at 64% correct classification accuracy, HRV alone at 56% accuracy and EEG alone at 62% accuracy (75). However, this study was conducted on a small population and thus requires further study in order to improve upon the application to classification. Gil et al. (97) used decreases in amplitude fluctuations of photoplethysmography (PPG) with an accuracy of 80%, sensitivity of 87.5% and specificity of 71.4%. Babaeizadeh and Zhou (98) created a novel method of ECG-derived respiration (EDR) combined with HRV for an accuracy of 88% and correct classification of 71%. Similarly, Lyons et al. (99) developed an ECG-derived respiratory power index (RPI) as an estimate for AHI to identify severe OSA in commercial drivers (Figure 2).

There are thus a variety of tools and combinations that appear to have potential in the detection and classification of apnea.

Collectively this body of studies points to the potential of diagnosing OSA *via* HRV parameters reflecting sympathetic hyperactivity during sleep, particularly during apneic episodes. However, more research needs to be done in this area as there are conflicting reports on the accuracy of using HRV alone, as compared to coordination with other measurements such as EEG, PPG, or EDR. Showing promise in the application of this idea, Le et al. (100) have made wearable device sensor technology predicting apneic episodes 1–5 min before onset with accuracy of 83.6, 80, 76.2, 66.9, and 61.1%, respectively, that could have many applications.

## HRV Changes During Arousal

Arousal interrupts sleep continuity to cause sleep fragmentation, which may contribute to cognitive impairment, excessive daytime sleepiness and adverse cardiovascular outcomes in OSA (101–103). Quantification of arousal would improve understanding of the underlying mechanism and relationship between arousal and OSA related outcomes (e.g., daytime sleepiness and functioning) (104, 105). Currently, EEG arousal is defined as the abrupt increase in high-frequency EEG activities lasting 3–15 s, following at least 10 s of sleep during NREM sleep. Additionally, increased chin EMG activity is needed during REM sleep according to the AASM criteria (106, 107). However, even if the concept of arousal should be extended, there currently is no agreement on the classification of arousal (108). Arousal could be divided into several states on the basis of specific causes. Two main types of arousal, physiologic (spontaneous or secondary to various stimuli), and pathologic (induced by sleep hypopnea and apnea, upper airway resistance syndrome or periodic limb movement) are commonly accepted (108, 109). Some studies tried to classify arousal manually based on whether an arousal is associated with a physiological event such as cortical arousal, respiratory arousal, cardiac arousal, movement arousal, snoring arousal, or SpO2 arousal (110, 111). It is reported that autonomic arousal does not have visual recognition in the way EEG arousal does. It is plausible that some peripheral stimulations may not be sufficient to lead to cortical visual EEG arousals but can cause cardiovascular perturbation (e.g., heart rate and blood pressure changes) (112–114).

In this case, autonomic arousal may be a new entity of arousals in OSA during sleep, possibly undetectable by EEG (115). Thirty percent of respiratory event termination causes are still undetermined. Some research indicates that it might be related to apnea-related autonomic arousal, which tends to be ignored due to its non-visible nature compared with other types of arousal in polysomnography (PSG) (116, 117). As a result, PSG would underestimate arousal severity if only visible EEG arousal counts. The occurrence of arousal induced by different causes varies in NREM and REM sleep (116). The underlying mechanisms between the central nervous system (CNS) and autonomic nervous system (ANS) in arousal is poorly understood. Arousal may be a contributor in cardiac alternations such as heart rate changes and blood pressure fluctuation. HRV changes accordingly since heart rate is accelerated and decelerated immediately pre- and post-arousal. Animal studies confirmed that transient arousal from NREM sleep is associated with acute cardiac sympathetic activation and parasympathetic withdrawal (118). The presence of arousal somehow immediately leads to wakefulness that differs in autonomic changes from rested wakefulness in other conditions (118).

Daytime cardiac vagal modulation improves due to the reduction of the frequency of arousals, suggesting arousal may trigger cardiac vagal inhibition. OSA is strongly related to hypertension, which is mainly attributed to sympathetic hyperactivity and/or vagal withdrawal causing a surge in heart rate and blood pressure during apnea-arousal episodes (119, 120). Study of autonomic arousals may help understanding why OSA patients with daytime sleepiness are associated with a higher risk of developing adverse CV outcomes such as hypertension and cardiac sudden death (121). It is reported that patients with co-morbid OSA and insomnia have a significantly higher number of arousals during sleep than OSA alone (122). Bennett et al. (123) found significant correlations between the autonomic arousal index based on pulse transit time analysis and pretreatment objective sleepiness (*r* = 0.49) and nCPAP responsive objective sleepiness (*r* = 0.44), suggesting autonomic arousal detection should be taken into account as a sleep fragmentation index to quantify sleepiness. Bartels et al. tried to define autonomic arousal. They found that lower blood pressure and high heart rate in the 15-s window before short-term cortical arousal and cardiovascular changes shift in the opposite direction after sleep recovery (110).

HRV provides insight to the processing of arousal response during sleep and improves the definition of arousal, criteria of detection and scoring, although it is still controversial. It should be included in the assessment of OSA for its useful clinical value. EEG arousal generally does not cause behavioral awakenings. However, arousal threshold measured by esophageal pressure, a gold standard for upper airway resistance syndrome, is invasive in clinical practice. On the other hand, cardiac arousal may reflect a neural response to stimuli. Little is known about the accumulation of persistent hyperarousal conditions in OSA. HRV would be a sensitive physiological index of autonomic arousal requiring more investigation. Further research is needed to understand the connectivity and interaction between the heart, its intrinsic nervous system, and the brain.

## Daytime Sleepiness and HRV

On the other end of arousal, daytime sleepiness, a multifactorial psychophysiological state, is one of the predominant symptoms in OSA (124, 125). Currently, the existing findings suggest that daytime sleepiness depends on the quantity and quality of prior sleep. Patients with OSA commonly suffer from reduced sleep quality that is related to fragmented sleep (126). Sleep disturbances caused by arousal are important contributors to sleepiness (123, 127, 128). Moreover, the frequency of arousal has more impact on sleep recovery than the amount of sleep (129). Subjective and objective sleepiness is often assessed by the Epworth sleepiness scale (ESS) and multiple sleep latency test (MSLT) (130, 131). ESS is a measure of a person's general daytime sleepiness, where a score ≥10 could be diagnosed as excessive daytime sleepiness. As a gold standard, the cut-off point of MSLT is still debatable based on the types of patients. According to the AASM, a sleep latency during MSLT of <8 min is defined as sleepiness. However, it is also suggested that mean sleep latency in MSLT <5 min is considered as pathological sleepiness, 5–10 min is suspected sleepiness, and 10–20 min is normal (130).

The prevalence of excessive daytime sleepiness (EDS) in OSA varies from 19 to 87.2% (132–134). However, 50% of individuals with moderate to severe OSA do not report EDS. Lombardi et al. (135) demonstrated that OSA patients with EDS had significantly lower baroreflex sensitivity and significantly higher low-to-high frequency power ratio of HRV during the different stages of nocturnal sleep compared to those without. Furthermore, subjects with EDS have a more blunted parasympathetic and more enhanced sympathetic cardiac drive during all sleep stages suggesting EDS is associated with cardiac autonomic imbalance. Guaita et al. (136) tested whether spectral and non-linear HRV help to differentiate sleep disordered breathing (SDB) patients with and without objective sleepiness, as assessed by the first 3 min of wakefulness during MSLT before sleep onset. Non-linear HRV (Correntropy) failed to detect sleepiness between groups.

However, some studies show that ESS increases with the severity of OSA (2, 137). EDS is not always related to AHI as a number of patients with moderate-to-severe OSA did not report subjective EDS in this evaluation (124, 135, 138). It raised the question of whether ESS is not adequately sensitive to detect sleepiness or if there are other underlying physiopathological mechanisms causing the development of sleepiness in OSA patients. Montemurro et al. (139) found severe OSA without EDS has higher very low frequency-HRV compared to those with EDS, indicating higher sympathetic heart rate control in sleepy patients. However, Sforza et al. (140) found that both diurnal and nocturnal time domain and frequency domain HRV failed to differ sleepy and non-sleepy elderly with unrecognized OSA according to ESS. Time with SaO2 <90% and total autonomic arousals were not significantly different between these two groups. Similarly, Bisogni et al. (141) reported that there is no correlation between EDS assessed by ESS and sympathetic activation in patients with mild to moderate OSA.

## HRV as a Risk Marker for Sleepiness Related Accidents

There is little doubt that attentional deficits affect driving capacity. Detection of drowsiness is importance in order to prevent road accidents due to SDB related sleepiness (142). Chua et al. (143) suggested that HRV has a strong association with psychomotor performance measured by psychomotor vigilance tests (PVT) to quantify vigilance performance in drivers. It is in line with previous studies using HRV in machine learning models to predict hypersomnolence in drivers with 90% accuracy (144–146).

It has been shown that sleepy OSA patients have a higher prevalence of adverse cardiovascular outcomes (e.g., hypertension) than non-sleepy OSA patients (147). Furthermore, excessively sleepy OSA patients are at increased risk of incident cardiovascular disease (CVD) compared to other OSA symptom subtypes (Disturbed Sleep, Minimally Symptomatic, and Moderately Sleepy) (121). However, ESS might not be reliable to evaluate the relationship between sleepiness and cardiovascular risk, a surrogate marker of sympathetic activity. MSLT is too time-consuming and costly to be a screening tool to score EDS. Given the association between sleepy OSA and cardiovascular disease has not been established, improving discrimination of sleepiness in OSA patients and the relationship between the severity of daytime sleepiness and HRV in larger-scale studies is required.

Previous studies have proven that CPAP could reduce daytime sleepiness (148). Less benefit from CPAP was found in OSA patients without symptoms than those with, suggesting treatments should be tailored (149–151). There are still 13% of patients with residual EDS after optimal CPAP treatment (152). They also found that the prevalence of residual excessive sleepiness was higher in moderate OSA than severe OSA, suggesting there is an underlying determinant contributing to EDS other than the severity of intermittent hypoxia and AHI. One of the possible determinants could be autonomic dysfunction during sleep. Abnormal autonomic regulation is also known to have an association with higher cardiovascular events in OSA. A possible relation between EDS and cardiovascular events in patients with OSA should be investigated in future studies (i.e., how autonomic dysfunction relates to the presence of EDS and contributes to its relevant consequences in these population).

## HRV Changes Due to Hypoxia

Exposure to hypoxia is a leading cause of oxidative stress, inflammation, and sympathetic hyperactivity (153). Recurrent oxygen desaturation induced by sleep apnea, one of the distinct features of OSA differing from non-OSA, may be associated with elevated sympathetic nervous activity and blood pressure (153). Additionally, Watson et al. (154) found that the severity of hypoxia is related to graded autonomic dysfunction. Both animal and human experiments demonstrated that the failure to restore cardiovascular adjustment capacity can be ascribed to impaired nerves and blunt responses of the autonomic system as a result of intermittent hypoxemia in OSA (155, 156). A systematic review shows that either SpO2 or SaO2 used to assess arterial oxygen saturation is correlated with time-frequency HRV during hypoxia in normal people at rest (157). Botek et al. (158) found lower arterial oxygen saturation (SpO2) in significantly reduced vagal withdrawal (Ln HF) and increased sympathetic-vagal balance, suggesting SpO2 level is related to the reaction of autonomic control to hypoxia. Their aim was to investigate if HRV could be used as a predictor of SpO2 response to hypoxic challenges in subjects normoxic at rest. Nevertheless, it is admitted that changes in detailed HRV parameters are not consistently similar due to the varying experimental protocols (e.g., the duration, severity, and types of hypoxia).

OSA generally generates a decrease in HRV during normobaric hypoxia in most reported investigations. However, there are still underlying complex central-peripheral interactions and modulation pathways in vulnerable populations. To address those issues, a growing body of studies have attempted to investigate the hypoxia burden in OSA (159–161). Time-dependent static and dynamic desaturation give more insight to the severity of hypoxia. Acute and chronic hypoxia may lead to different autonomic modulation mechanisms. Hypoxia activated chemoreflex leads to acutely increased short-term sympathetic tone during the occurrence of sleep apnea (54). Furthermore, hypoxia exerted long-lasting chronic effects during the daytime and impaired baroreflex sensitivity (162). Meanwhile whether or not sympathetic hyperactivity induces parasympathetic inhibition is still controversial. The overall reduced HRV with increased sympathetic tone resulted from chronic hypoxia, while a rise in HRV with decreased vagal withdrawal occurred due to the subsequent adaptation and improved tolerance to short-term exposure to repeated hypoxic stress (163). Geovanini et al. demonstrated a vicious circle between hypoxia-induced inflammation and cardiac autonomic abnormality with elevated sympathetic or reduced parasympathetic tone. They also found the values of SDNN, LF, and HF are closely linked to OSA severity while only mean heart rate significantly correlated with augments in neutrophils (164). In an OSA children study, Walter et al. (165) found that OSA may have negative influence on cerebral blood flow due to the attenuated central autonomic control by mediating HRV. Therefore, it is reasonable to believe that different cardiac autonomic modulation responses occur either due to reduced vagal modulation, sympathetic predominance, or even a combination of these responses.

The possibility of an increasing risk in the mortality and morbidity in hypoxic OSA patients with autonomic dysfunction requires further evidence. In addition, both hypoxia and arousal have confounding effects on respiratory-cardiac coupling. Which one is the determinant of cardiac autonomic dysfunction in OSA is controversial in animal and human studies (5). It seems that in prospective animal studies, OSA-induced hypoxia has a persistent impact on daytime hypertension compared to acoustic arousal-induced control models, which exerted nocturnal elevations in blood pressure. However, in humans, the answer to that question is uncertain. Norman et al. suggested that CPAP therapy, which reduced both the intermittent hypoxia and arousals, plays a more important role in improving cardiovascular autonomic function than elimination of nighttime intermittent hypoxia by comparing the results of 24-h ambulatory blood pressure in moderate-to-severe OSA patients who received either CPAP therapy or sham-CPAP with supplemental oxygen (166).

Some studies indicated that certain damages of autonomic function are reversible after eliminating physiological influences (e.g., arousal, hypoxia, and respiratory events) in OSA population with CPAP treatment (81, 167, 168). Thus, HRV maybe become a potential early indicator of the adverse effects of hypoxia on OSA and identifying treatment responses. To date, the effect of nocturnal hypoxia on HRV patterns is unknown and correlation studies of HRV and hypoxia in HRV are limited. Those results may contribute to monitoring the progress of chronic sustained normobaric hypoxia on the cardiovascular and autonomic systems.

## HRV in Pediatric OSA

OSA affects 0.1–13% of children, particularly occurred in pre-school age (169). Pediatric OSA characterize by prolonged partial OSA, which usually occurred in REM sleep, preserved sleep architecture, uncommon OSA-related cortical arousals and recurrent hypoxia (170). Enlarged tonsils and adenoids are the leading causes of OSA in children. Unlike adult OSA manifested with excessive daytime sleepiness and cognitive dysfunction, pediatric OSA is more likely to have negative impact on the development of the central nervous system and cardiovascular system, potentially leading to neurobehavioral deficits (e.g., growth impairment, behavioral, and learning problems) (171). Overt cardiovascular disease is not common in pediatric OSA compared to adults (172), but early evidence shows that pediatric OSA is related to left ventricular hypertrophy (173, 174), abnormal blood pressure fluctuation (175, 176), and reduced systolic and diastolic function (177, 178). HRV analysis is increasingly explored in assessment for cardiovascular autonomic control, the screening and diagnosis of sleep apnea and efficacy of treatments in pediatric OSA during daytime and nighttime due to its feasibility (179, 180). Current findings suggested that altered HRV patterns during daytime and sleep are also found in childhood OSA (181). Not surprising, there are more discrepancies in the results of frequency domain analysis than in time domain analysis due to diverse subject samples and the different methodologies (182, 183). Chaicharn et al. (179) tried to quantify daytime autonomic function in non-OSA and OSA children with spectral HRV analysis, showing OSA children have significant elevated sympathetic tone but normal parasympathetic control, with less reactive response to autonomic tests compared to controls. Liao et al. (182) found autonomic imbalance with increased LF/HF during sleep among groups with different levels of AHI. Similarly, Baharav et al. (183) were able to show sympathetic augmentation with increased LF both during wake before sleep onset and during sleep. By contrast, Kwok et al. (180) demonstrated no changes in most of the important time-domain and HRV measures between non-OSA and OSA children using 1-h ECG data. Impaired baroreflex adaptation is also found in OSA as it is associated with a decrease in nighttime baroreflex gain (184). Autonomic activity may play a key role in pharyngeal compliance of childhood OSA (185). Another application of HRV in childhood OSA is to evaluate treatment response. Muzumdar et al. (186) reported that HRV improved with decreased sympathetic and increased vagal tones after adenotonsillectomy in children with OSA, while no changes showed in HRV in moderate-severe pediatric OSA with 1-year non-invasive ventilation (187). The results of long-term effect of OSA on HRV are debated. Vlahandonis et al. (188) failed to show significant differences in autonomic regulation determined by using HRV analysis among children with habitual snoring, and those with and without OSA regardless of intervention during 4-year follow-up visits. However, Walter et al. (189) found improved HRV in preschool-aged children with resolved OSA, showing decreased LF and HF, while increased HF in those with unresolved OSA during 3-year period. It is noteworthy that age, obesity, sleep stage, and AHI severity are independently correlated with HRV measurements in children (190, 191). Explanations on these results need to be cautious with those confounding factors. Whether or not HRV measures could be the reliable maker of disease severity and risk stratification in children with OSA is still unproven. The clinical implication of cardiac autonomic alternation in pediatric OSA and how it disrupts the maturation of autonomic control and affects the nervous and cardiovascular functioning need further investigation.

## Effect of Age, Ethnicity and Sex on HRV

Previous studies have demonstrated age-, ethnicity-, and sex-specific differences in HRV in the healthy general population and under certain conditions. It is generally accepted there is an inverse association between age and HRV. However, it is unclear whether the effects of OSA on cardiac autonomic modulation in elderly subjects (>60 yr) are different from those in other age groups (young and mid-aged adults). Trimer et al. compared the differences in HRV among the elderly and the young population with and without OSA. They found the elderly with OSA have significantly lower LF/HF ratio only during wakefulness at night than the young with OSA but not during other sleep stages (192). Sforza et al. (140, 193) suggested age may have more devastating effect on HRV in the elderly, which possibly undermines the application of HRV in those population.

Findings on sex and ethnic differences in HRV are less consistent (194). Nonetheless, reduced HRV is related to higher cardiovascular morbidity and mortality, where decreased cardiac vagal control is considered an important contributor. Currently, a majority of studies report females are characterized by higher vagal control assessed by HF-HRV and lower sympathetic control assessed by LF-HRV (194). Furthermore, women exhibit more complex heart rate dynamics (195). Several studies found no difference between men and women in HF-HRV or that men have a higher HRV (196–200). These results contradict previous findings that women are less likely to develop progressively cardiovascular diseases compared to men (201, 202).

In terms of interaction associations between HRV and age, sex, and ethnicity, Liao et al. (203) found changes in autonomic function have close associations with age, ethnicity, and gender in a community-based cohort by spectral analysis of HRV. They found that the sympathetic and parasympathetic tone decrease with increasing age in a general population. White populations have a higher LF, HF, and lower HF/LF than black populations, suggesting that white populations show sympathetic predominance in cardiac regulation. Men have a higher LF, and a lower HF/LF ratio than women. Those results demonstrate white and male populations have higher sympathetic activity, which is considered as a major contributor to cardiovascular diseases (e.g., hypertension). In contrast, Sloan et al. (200) reported that there is a higher standard deviation of RR intervals in white subjects compared to black subjects, and in men compared to women with age between 33 and 47 years old. No ethnicity- and sex- special differences were found in HF-HRV. Comparatively, Choi et al. found significant ethnically related differences and age-related differences (in Caucasian Americans but not in African Americans) in short-term daytime spectral HRV. Young African Americans showed a similar HRV profile to older Caucasian Americans, leading Choi et al. (204) to suggest the presence premature autonomic nervous system aging in young African Americans. A few studies related to those correlates on HRV during sleep are available. Hall et al. suggested that ethnicity is associated with HRV during sleep. They found white women have decreased parasympathetic tone and elevated sympathetic tone during NREM stage 2 and REM sleep compared to their African American and Chinese counterparts after controlling for confounding factors such as recording length and respiratory rate (205). Huang et al. (206) have shown heart rate profiles in a larger cohort of adults without sleep apnea in order to develop heart rate phenotypes regarding sleep physiology. They implied that heart rate dipping and spectral HRV metrics could contribute to sleep phenotyping due to their significant correlations to sleep measures (e.g., sleep stage, total sleep time and sleep quality). Interpreting the clinical relevance between ethnicity, sex, and HRV should be approached with caution due to the plethora of confounding factors, such as physiological, psychological, behavioral, and sociodemographic factors. To date, there is limited data reporting on the influence of age, ethnicity, and sex on HRV in the OSA population. It is unclear which OSA phenotypes are most likely to develop cardiovascular diseases and thus, which patients are most likely to benefit from CPAP or other forms of therapy for OSA (207). Those findings in cardiac heterogeneity might lead to a better understanding of the underlying cardiovascular pathophysiology and cardiovascular risk stratification in patients with OSA. Additionally, it would facilitate the development of effective strategies for treatment decision of OSA according to cardiac phenotypic characterization in order to improve treatment efficacy and predict treatment outcomes.

## HRV and OSA Comorbidity with Psychiatry Diseases

Psychophysiological disturbances have significant impacts on the autonomic nervous system (ANS) (3, 208–211). Depression and anxiety are considered as psychosocial risk factors for cardiovascular comorbidity (212). HRV analysis has been used to quantify autonomic dysregulation in insomnia, depression, anxiety, and schizophrenia (208, 211). Epidemiological data has shown that 39–58% of patients with insomnia and 5–63% of patients with depression had accompanying OSA diagnoses (213–215). Additionally, it was found that co-morbid OSA and insomnia patients are at a higher risk of developing psychiatric disorders such as anxiety and depression than OSA patients without insomnia (122, 216). Interestingly, OSA and insomnia are more likely to show opposing clinical symptoms related to sleepiness and alertness (215). Nevertheless, increased sympathetic activity and depressed parasympathetic activity were exhibited both in OSA and insomnia (25, 217). It is reported that untreated OSA aggravate insomnia in the disturbed sleep cluster due to hyperarousal (218). Augmentation in heart rate and sympathetic tone, which is thought to be essential to the alertness and motivation, may play a key role in the pathophysiology of insomnia (215). However, interaction mechanisms between OSA and insomnia of autonomic control evaluated by HRV measures remain unclear.

Reduced global HRV is consistently reported in depression and anxiety disorders. Specifically, depression is characterized by increased cardiac rhythmicity and reduced heart rate variability during both sleep and wakefulness (219). Moreover, changes in HRV parameters are associated with alternations in symptom severity of depression (220). Saad et al. (219) showed that a sleep heart rate profiling algorithm detecting whether individuals with sleep complaints experience depression has an identification accuracy of 79.9%. Similarly, anxiety disorders displayed significantly lower HRV (221). Recently, two reviews highlighted the wide applications of HRV in mental health and psychiatric disorders (221). Likewise in populations under 18 years old, there was evidence implied that a resting state measure of HF-HRV is associated with depressive symptoms in children and adolescents with depression (222). In combination with functional brain imaging, HRV mediated by the prefrontal cortex may provide evidence of heart-brain network response to stressors and stimuli to maintain homeostasis (9). Unfortunately, co-morbid psychiatric symptoms and disorders in OSA are often ignored or misdiagnosed. Only a paucity of studies has been reported to investigate ANS dysregulation in OSA populations concomitant with psychiatric conditions *via* HRV analysis.

Evidence of autonomic dysfunction in OSA with various psychiatric and psychological disorders deepens the understanding of their psychopathology and physiopathology associated with negative cardiovascular outcomes. Correlation studies of OSA and neuropsychiatric diseases in ANS function assessed by HRV are lacking. Furthermore, it would be challenging to diagnose and treat co-morbid psychiatry disorders and OSA. It is known that the administration of drugs for psychiatric treatment aggravates OSA as it potentially reduces upper airway muscle tone to impair airway stability, decreases ventilatory response to hypoxia, increases arousal threshold leading to prolongation of respiratory events and deteriorates oxygen saturation. It seems that HRV analysis could be highly applicable in the exploration of the cardiovascular and psychopathological implications in psychiatric disorders. Investigations in the overlapping conditions in physiological and psychological aspects in OSA patients who have worse clinical outcomes and treatment response are warranted. Quintana et al. (223) provide guidelines and recommendations to advance heart rate variability research in psychiatry. We expect more perspectives and possible application of HRV in OSA in neuropsychiatric alternations could be discussed in future studies.

## HRV and Cardiovascular Mortality and Morbidity

Due to HRV being a marker of autonomic innervation of the heart, it has been suggested that increased sympathetic activity during sleep due to OSA may be a link to cardiovascular disease (54). Sympathetic dominance during sleep has been shown in those with ischemic heart disease (53), coronary artery disease (CAD) (55) and post-MI (224). Consequently, HRV parameters are markers for adverse CVD prognoses (49, 51, 52).

Several cardiovascular disease studies have reported an increased risk of mortality in relation to altered HRV parameters. Kleiger et al. found that a 24-h SDNN of <50 ms carried a relative risk of mortality 5.3 times higher than an SDNN of over 100 ms. They suggested that increased sympathetic or decreased vagal tone may predispose to VF (51). Zemaityte et al. (53) found that increased LF and decreased HF was related to the degree of deterioration of IHD functional state in overnight HRV analysis. Post-MI there is a lack of NREM vagal activity that is more likely to lead to lethal arrhythmic events and sudden death (224). Kearney et al. (49) reported that those with chronic HF and 10% lower SDNN had a hazard ratio of 1.06. Rich et al. found that EF and decreased HRV were the best predictors of 12-month mortality post-coronary angiography without recent MI. The HRV contribution to mortality was found to be independent of other disease-related variables, and the 12-month mortality was 18 times higher in those with an HRV <50 ms (52). To further this, Mäkikallio et al. (225) found that random elderly patients with altered HRV parameters predicted a 2.5 relative risk of cardiac death and 4.1 for sudden cardiac death. Algra et al. (226) found that low SDNN was correlated with a 2.6-fold risk of sudden death, also adding that low parasympathetic activity is a risk factor for sudden death. The correlation between altered HRV parameters reflective of dysfunctional sympathovagal balance and increased mortality risk is thus well-established in CVD.

Additionally, CVD and OSA have been shown to be linked (227–234). The Sleep AHEAD study found a greater prevalence of stroke at greater AHI but no association between CHD and OSA (235). However, there were very few patients with CHD and thus the concluded relationship is not a representative analysis of an OSA association with coronary heart disease (CHD). Gottlieb et al. (233) found that OSA was a predictor of incident heart failure with an adjusted hazard ratio of 1.13 per 10 unit increase in AHI. In a meta-analysis of 25,760 subjects, Wang et al. (236). found that severe OSA significantly increases CVD risk, stroke and all-cause mortality with relative risks of 1.79, 2.15, and 1.92, respectively. A positive association was found between moderate OSA and CVD but not with mild OSA. A 10 unit increase in AHI was associated with 17% greater risk of CVD. No correlation was found between CHD and OSA, but again the number of prospective studies relating CHD and OSA were limited and lacked power for definitive conclusion (236). Yaggi et al. (232) found that OSA independently increases risk of stroke and all-cause mortality with a hazard ratio of 1.97 post-adjustment. In a systematic review, Lavie (234) also concluded that sleep apnea is a mortality risk that can be reduced *via* CPAP, which is especially crucial in younger patients, as they carry a higher mortality risk.

In the linking of OSA and CVD, HRV, and OSA mortality and mortality, the exact physiological pathway through which these are connected is not well-understood. In animal models, Iturriaga (237) proposed that intermittent hypoxia induces carotid body potentiation, and that current evidence indicated that this alters the sympathetic, vascular, and ventilatory response to hypoxia. Whether this is the exact mechanism and whether it increases CVD risk is not definitively known. However, repetitive oxygen desaturation episodes are associated with HRV parameters suggestive of cardiac sympathetic predominance. In a group of CAD patients, those with LVEF >50% had a higher LF:HF ratio than those with LVEF ≤ 35% during cyclic oxygen desaturation episodes but not during control episodes (55). This suggests that hypoxia worsens pre-existing cardiovascular conditions. A few results of the secondary analyses using ECG data from the Sleep Heart Health Study (SHHS) or the Wisconsin Sleep Cohort Study are reported. Bradicich et al. (238) and Wang et al. (24) demonstrated associations between HRV and characteristics of polysomnographic parameters, however, they did not attempt to use HRV as a CVD risk predictor in this part of the SHHS dataset. Sankari et al. (239) suggested beat-to-beat intervals index (RRDI) during sleep is closely correlated to new-diagnosis CVD (hazard ratio of 1.21 per 10-unit increment in RRDI) in OSA patients from the Wisconsin Sleep Cohort, but they did not utilize other linear and non-linear HRV measures to show the further relationship between CV risk and OSA.

However, despite the clear association between OSA and mortality and CVD, more studies need to be done to determine the exact physiological mechanisms by which this occurs, and if OSA is an independent causal factor of increased mortality and CVD risk as suggested. From the current data, altered HRV features such as SDNN are good predictors of cardiovascular mortality. There appears to be a correlation of higher mortality risk and lower SDNN, but the cut-off point varies depending on the populations and the length of ECG segments. Therefore, determination of a clear cut-off value of SDNN requires further investigation (240).

## Conclusion

With more sophisticated analytical approaches and techniques developing, HRV measures could provide additional electrophysiological information on impaired cardiovascular alternation, which might be related to subclinical cardiovascular outcomes in patients with OSA. It is already known that the determination of time window (ECG segment length and SDB-related events) is critical to HRV analysis, but a standardized analytical approach is lacking. HRV is proving to be accurate in sleep staging and particularly screening and diagnosing OSA. However, a combinatorial method of HRV and EDR provides hidden information on cardiopulmonary coupling, which transfers from heart rate to respiration and improves the accuracy of sleep apnea detection compared to either method alone. The cognitive consequences and the daytime outcomes of ANS alternation during sleep in patients with OSA are unclear. The use of HRV in the prognosis of OSA independent of CVD is also unclear. However, HRV has shown a close association to mortality and co-morbidities. Additionally, overlapping conditions increase progressively in OSA, requiring reliable tools to manage those conditions at an early stage. Further studies are required to explore the implications of integrated cardiac physiology in regulatory networks between the central brain and heart. In particular, following this investigation, several research topics have been found to be of value:

- Prospective studies using HRV to accurately predict cardiovascular outcomes in OSA should be as a priority for clinical application of HRV research
- Studies investigating cardiac OSA phenotypes on the basis of HRV profiles to facilitate the definition of OSA subtypes and implement tailored treatment approaches in clinical practice
- New sophisticated methods of HRV analysis to analyze the inevitable instationarities of OSA's transitional nature that prove challenging for current algorithms and models
- Context-dependent analyses of HRV (i.e., age, BMI, gender, sleep stages) to better understand the association between anthropometric and sleep characteristics and autonomic function in OSA
- Investigation and standardization of the time window segments analyzed to provide comparable and valuable ECG data in OSA during an overnight sleep study

HRV is showing promise in clinical application and due to the already large and increasing prevalence of OSA, these further studies are imperative to the advancement of diagnostic and treatment approaches needed to minimize the existing and future health and financial burden.

## Author Contributions

HQ and TP were responsible for the manuscript concept and design. HQ, NS, and TP prepared the manuscript draft. JFK prepared the figures. HQ, NS, TP, MG, NW, JFK, and FV-V contributed to critical revision of the manuscript. All authors contributed to the article and approved the submitted version.

## Conflict of Interest

The authors declare that the research was conducted in the absence of any commercial or financial relationships that could be construed as a potential conflict of interest.
